# Supplementary material for: Comparison of three reconstruction algorithms for low‐dose phase‐contrast computed tomography of the breast with synchrotron radiation
Source: Med Phys. 2025 Jul 15;52(7):e17950. doi: 10.1002/mp.17950 (PMC12260779; doi:10.1002/mp.17950)
Supplement: Supplementary file 1 — Supporting Information [file MP-52-0-s002.pdf]

SUPPLEMENTARY TABLE 1. Objective measurements results for the thin coronal slices.

**Sample Number**	**Contrast (Dimensionless)**			**SNR (Dimensionless)**			**CNR (Dimensionless)**			**Res (pixels)**			**Q_s (pixels$^{-1.5}$)**		
	FBP	*UTR*	*cSART*	*FBP*	*UTR*	*cSART*	*FBP*	*UTR*	*cSART*	*FBP*	*UTR*	*cSART*	*FBP*	*UTR*	*cSART*
1	0.38 ± 0.05	0.31 ± 0.03	0.33 ± 0.04	5.11 ± 0.60	6.36 ± 0.92	10.53 ± 1.34	2.25 ± 0.08	2.31 ± 0.08	3.94 ± 0.19	1.98 ± 0.04	1.76 ± 0.05	1.98 ± 0.13	1.90 ± 0.12	2.88 ± 0.41	3.80 ± 0.07
2	0.36 ± 0.02	0.31 ± 0.02	0.33 ± 0.02	8.58 ± 2.98	10.58 ± 3.04	11.14 ± 3.63	3.44 ± 0.46	3.61 ± 0.35	5.27 ± 0.49	1.88 ± 0.15	2.04 ± 0.11	1.74 ± 0.15	3.71 ± 1.61	4.10 ± 1.61	5.53 ± 2.52
3	0.38 ± 0.038	0.33 ± 0.03	0.34 ± 0.04	6.73 ± 1.01	8.60 ± 0.68	12.22 ± 2.24	1.90 ± 0.13	2.33 ± 0.20	2.96 ± 0.34	2.00 ± 0.17	2.18 ± 0.05	1.89 ± 0.12	2.41 ± 0.02	2.80 ± 0.29	4.77 ± 0.31
4	0.37 ± 0.05	0.30 ± 0.04	0.30 ± 0.04	5.39 ± 0.33	8.35 ± 0.17	10.06 ± 0.46	2.19 ± 0.52	2.74 ± 0.57	3.52 ± 0.71	1.86 ± 0.17	2.32 ± 0.07	1.99 ± 0.09	2.17 ± 0.39	2.52 ± 0.77	3.78 ± 1.12
5	0.36 ± 0.02	0.31 ± 0.02	0.32 ± 0.02	6.01 ± 1.06	9.21 ± 1.04	11.87 ± 2.35	2.62 ± 0.61	3.17 ± 0.49	4.58 ± 1.4	1.92 ± 0.14	2.39 ± 0.09	2.09 ± 0.35	2.34 ± 0.24	2.59 ± 0.22	4.16 ± 0.22
6	0.36 ± 0.01	0.31 ± 0.01	0.32 ± 0.01	4.76 ± 1.32	6.35 ± 1.19	8.73 ± 2.32	1.81 ± 0.15	2.19 ± 0.18	3.19 ± 0.16	1.69 ± 0.15	1.94 ± 0.04	1.79 ± 0.19	2.17 ± 0.33	2.48 ± 0.77	3.82 ± 0.96
7	0.38 ± 0.02	0.32 ± 0.02	0.34 ± 0.02	6.86 ± 2.07	9.33 ± 2.01	12.78 ± 3.29	2.09 ± 0.45	2.44 ± 0.37	3.06 ± 0.60	1.98 ± 0.24	1.81 ± 0.05	1.90 ± 0.26	2.47 ± 0.29	3.94 ± 0.69	4.97 ± 0.24
8	0.34 ± 0.02	0.30 ± 0.03	0.30 ± 0.01	5.92 ± 0.91	7.51 ± 1.00	9.30 ± 1.95	1.84 ± 0.41	2.26 ± 0.42	2.61 ± 0.39	2.14 ± 0.06	2.16 ± 0.03	1.97 ± 0.13	1.96 ± 0.28	2.50 ± 0.38	3.52 ± 0.49
9	0.36 ± 0.01	0.33 ± 0.02	0.33 ± 0.02	5.93 ± 1.15	7.70 ± 1.45	10.52 ± 3.16	2.38 ± 0.36	2.90 ± 0.18	4.07 ± 0.59	2.11 ± 0.12	2.04 ± 0.16	2.03 ± 0.14	1.96 ± 0.20	2.80 ± 0.04	3.78 ± 0.06
10	0.36 ± 0.03	0.29 ± 0.04	0.32 ± 0.03	6.68 ± 1.22	9.32 ± 1.56	11.97 ± 4.41	3.03 ± 1.72	3.57 ± 1.88	4.00 ± 2.42	2.19 ± 0.11	2.31 ± 0.10	1.96 ± 0.26	2.09 ± 0.36	2.72 ± 0.33	4.44 ± 0.80

Results are reported as average value (evaluated across three different slices) plus/minus the standard deviation.
