# Supplementary material for: Comparison of three reconstruction algorithms for low‐dose phase‐contrast computed tomography of the breast with synchrotron radiation
Source: Med Phys. 2025 Jul 15;52(7):e17950. doi: 10.1002/mp.17950 (PMC12260779; doi:10.1002/mp.17950)
Supplement: Supplementary file 2 — Supporting Information [file MP-52-0-s001.pdf]

SUPPLEMENTARY TABLE 2. Objective measurements results for the thick axial slices.

Sample Number	Contrast			SNR			CNR			Res			Q_s		
	FBP	*UTR*	*cSART*	*FBP*	*UTR*	*cSART*	*FBP*	*UTR*	*cSART*	*FBP*	*UTR*	*cSART*	*FBP*	*UTR*	*cSART*
1	0.23 ± 0.01	0.23 ± 0.01	0.19 ± 0.01	6.05 ± 0.87	5.66 ± 0.83	11.45 ± 2.64	1.38 ± 0.19	1.30 ± 0.19	2.18 ± 0.56	2.05 ± 0.01	1.72 ± 0.01	2.04 ± 0.04	2.06 ± 0.28	2.51 ± 0.37	3.91 ± 0.79
2	0.19 ± 0.03	0.17 ± 0.03	0.17 ± 0.03	5.31 ± 0.14	6.57 ± 0.21	7.65 ± 0.25	1.03 ± 0.16	1.11 ± 0.25	1.30 ± 0.25	2.23 ± 0.02	2.07 ± 0.02	1.82 ± 0.01	1.59 ± 0.06	2.21 ± 0.10	3.12 ± 0.09
3	0.24 ± 0.02	0.21 ± 0.02	0.20 ± 0.01	6.01 ± 0.34	7.20 ± 0.17	9.31 ± 0.76	1.45 ± 0.10	1.49 ± 0.12	1.86 ± 0.11	2.09 ± 0.03	2.01 ± 0.1	1.83 ± 0.02	1.99 ± 0.08	2.53 ± 0.05	3.75 ± 0.26
4	0.19 ± 0.02	0.17 ± 0.02	0.16 ± 0.01	5.13 ± 0.35	6.86 ± 0.41	8.46 ± 0.64	0.97 ± 0.18	1.21 ± 0.21	1.36 ± 0.22	2.16 ± 0.01	2.07 ± 0.02	1.90 ± 0.02	1.62 ± 0.12	2.31 ± 0.15	3.22 ± 0.21
5	0.22 ± 0.02	0.20 ± 0.01	0.19 ± 0.01	5.22 ± 0.43	7.17 ± 0.43	8.43 ± 1.13	1.17 ± 0.06	1.42 ± 0.08	1.58 ± 0.13	2.13 ± 0.04	2.01 ± 0.03	1.70 ± 0.03	1.68 ± 0.12	2.51 ± 0.13	3.79 ± 0.42
6	0.21 ± 0.03	0.20 ± 0.03	0.19 ± 0.03	4.57 ± 0.70	5.10 ± 0.67	6.86 ± 1.75	0.96 ± 0.10	1.02 ± 0.10	1.32 ± 0.27	2.18 ± 0.05	1.98 ± 0.02	1.70 ± 0.03	1.42 ± 0.18	1.83 ± 0.21	3.08 ± 0.70
7	0.23 ± 0.04	0.19 ± 0.04	0.20 ± 0.03	5.43 ± 0.52	5.70 ± 0.68	8.99 ± 1.25	1.22 ± 0.12	1.09 ± 0.12	1.77 ± 0.13	2.15 ± 0.02	1.90 ± 0.01	1.73 ± 0.02	1.72 ± 0.14	2.18 ± 0.27	3.96 ± 0.51
8	0.20 ± 0.01	0.18 ± 0.02	0.18 ± 0.03	6.09 ± 0.33	6.69 ± 0.33	7.33 ± 0.91	1.23 ± 0.02	1.23 ± 0.28	1.33 ± 0.22	2.14 ± 0.01	1.92 ± 0.01	1.90 ± 0.01	1.95 ± 0.10	2.51 ± 0.14	2.80 ± 0.35
9	0.20 ± 0.01	0.19 ± 0.02	0.19 ± 0.01	5.96 ± 0.48	6.29 ± 0.47	8.35 ± 1.91	1.17 ± 0.11	1.18 ± 0.13	1.61 ± 0.35	2.37 ± 0.04	1.91 ± 0.01	1.87 ± 0.03	1.63 ± 0.09	2.38 ± 0.16	3.26 ± 0.69
10	0.21 ± 0.02	0.20 ± 0.02	0.20 ± 0.01	5.94 ± 0.34	7.20 ± 0.37	8.34 ± 1.66	1.24 ± 0.04	1.44 ± 0.03	1.69 ± 0.03	2.32 ± 0.14	2.25 ± 0.12	1.74 ± 0.01	1.69 ± 0.12	2.14 ± 0.17	3.62 ± 0.68

Results are reported as average value (evaluated across three different slices) plus/minus the standard deviation.
